# Government regulation strategy, leading firms’ innovation strategy, and following firms imitation strategy: An analysis based on evolutionary game theory

**DOI:** 10.1371/journal.pone.0286730

**Published:** 2023-06-08

**Authors:** Mengke Zhang, Yan Huang, Yifan Jin, Yuan Bao

**Affiliations:** School of Management, Beijing Union University, Beijing, China; Shanxi University, CHINA

## Abstract

In the innovation ecosystem, the knowledge-based game behavior of each subject not only pertains to its own survival and development but also affects evolution of the innovation ecosystem. The present study investigates the choice of government’s regulation strategy, leading firms’ innovation protection strategy and following firms’ imitation strategy from the perspective of group evolutionary game. Based on the cost-benefit perspective, an asymmetric tripartite evolutionary game model and a simulation model are constructed to analyze the strategies and stability of the evolutionary equilibrium of each subject. We focus mainly on the protection intensity of innovation achievements by leading enterprises and the difficulty of imitation and substitution by following enterprises. The cost of patent operation and maintenance, government subsidies, and the relative difficulty of technology substitution and imitation were identified as the key factors affecting the evolutionary equilibrium of the system. Based on different scenarios resulting from the aforementioned factors, four equilibrium states are observed in the system, namely {no government regulation, technology secrecy, substitution}, {no government regulation, technology secrecy, imitation}, {no government regulation, patent application, imitation}, and {government regulation, patent application, imitation}. Finally, the study suggests corresponding recommendations for the three parties, which can help governments as well as the leading and following firms to choose appropriate behavioral strategies. At the same time, this study offers positive insights to participants in the global innovation ecosystem.

## Introduction

Under the new economic normal, innovation ecosystems are a crucial factor for supporting national economic transformation and high-quality development. Innovation ecosystems are self-adaptive, self-organizing, and self-coordinating synergistic evolutionary complex systems that promote diverse ecological niches and dynamic interactions among populations [1]. Along with the development of the global digital economy, the issue of protection of innovation has become increasingly intense and involves a wide range of industries. Considering the smartphone innovation ecosystem as an example, companies such as Apple and Qualcomm reap the advantages of technological innovation and are among the core leading enterprises in the innovation ecosystem, while companies such as Samsung, Huawei, and ZTE are the following enterprises. Under the patent protection policy and subsidies introduced by the government, the leading companies seek a balance between protecting the patent system with disclosure and maintaining the leading edge through technology secrecy. Additionally, some organizations focus on protecting innovation with patents, such as Qualcomm’s patent barriers in wireless communication and chip design, while some adopt technology secrets to prevent tacit knowledge spillover, such as Apple with its operating system. Samsung and other following companies learn from the leading companies, with some choosing imitation strategies to catch up quickly and some seeking differentiated positioning through alternative strategies. However, there is another phenomenon, that is, the government does not have a clear penalty for excessive imitation, and many small smartphone companies imitate features of Apple products such as appearance to deceive consumers and disrupt the market order. Knowledge-based competition is the main feature of this innovation ecosystem, and dynamic games among the subjects drive the development of the smartphone innovation ecosystem. The strategic choice of innovation ecosystem subjects is a common concern in the theoretical and practical communities, which is the core issue explored in this study.

Evolutionary game theory provides a powerful framework for research related to the strategic choices of subjects in innovation ecosystems [2]. Scholars have identified several typical factors that enhance inter-subjective interactions [3], such as kin selection [4], direct or indirect reciprocity [5], group selection [3] and spatial reciprocity, all of which strongly favor the persistence and emergence of altruistic behavior. A study indicated that evolutionary cooperation on interdependent systems has become an active and challenging issue [6]; specifically, cooperation on interdependent networks dominated by firms and governments has increased greatly in recent years [7]. In terms of population distinction, an innovation ecosystem can be considered a collection of three populations, namely government, leading firms, and following firms, all of which influence and constrain each other and form the core kinetic energy that drives evolution of the innovation ecology [8]. Scholars have comprehensively studied governments and firms and found that they play different roles in the innovation ecosystem and that their behavioral strategies are influenced by different factors. Leading firms, as the main body of independent innovation, guide ecological evolution and are the driving force of supply-side structural reforms [9]. Following firms play a crucial role in balancing supply and demand, diversification of ecological niches, and growth and evolution of innovation ecosystems, and they are the cornerstones for promoting upgrades and industrial development improvement in innovation ecosystems [10, 11]. The government-regulated intellectual property rights (IPR) system constitutes an institutional framework that ensures not only the balance between the leading and following firms but also sustainable development of the innovation ecosystem. Due to the spillover risk of innovation [12], there is usually a trade-off between patents and technology secrets in leading firms [13–15], which helps protect innovation and sustain competitiveness. Following firms often choose imitation or alternative strategies to catch up based on the perceived benefits and risks of the IPR regime and technological environment [16, 17]. Government regulation is typically considered an effective policy tool for overcoming market failure and promoting sustainable economic growth [18]. It helps ensure stable development of the innovation ecosystem. The innovation protection strategy of leading firms and the following strategies of the following firms result from a dynamic game among the three innovation ecosystem subjects under the government-regulated IPR system framework. Organic linkages and dynamic feedback among the main strategies promote synergistic evolution of the innovation ecosystem [19].

Although previous studies have revealed the organic linkage between government and firms, some shortcomings remain to be addressed, which are as follows: first, previous studies have focused on information disclosure and protection role of the patent system, ignoring the impact of the patent application subsidy mechanism on firms’ innovation protection strategies, and this shortcoming is especially prominent in the context of emerging economies. Second, previous studies have mainly considered the game between government and firms or between firms, while the strategies of subjects in the innovation ecosystem are formed and evolved in the group ecology, with heterogeneity among firms. Third, previous studies have focused mainly on the strategies of leading firms, ignoring the mechanism of strategy choice between government decisions and following firms in the innovation ecology and its impact on the development of innovation ecology. In addition, we consider the following issues in the present study: first, due to the different development backgrounds and management habits of firms, they are prone to adopt different innovation strategies, leading to a wide range of changes in decision-making behavior and simultaneous occurrence. Among them, the subjects in the game process should take various actions to approach the actual practice. Second, we should not assume that all subjects are completely rational in their decision-making. Making an informed choice among the many possible innovation strategies is challenging because following firms may be blind to government regulation strategies as well as to the innovation behavior chosen by leading firms. Finally, we should consider that in a real innovation ecosystem, the final balanced decision outcome may not be the most beneficial for each subject, and each subject will be influenced by each other’s decisions to adjust their own decisions to ensure minimum loss.

Based on the aforementioned deficiencies and discussions, the practical application of the current common practice of estimating innovation ecosystems is still a long way. The subjects of innovation ecosystems should adopt a dynamic and diverse set of strategies. Therefore, this study primarily aimed to construct an evolutionary game model of the government and the leading and following firms, as well as to investigate the impact of tripartite behavior on the innovation ecosystem. First, we integrated the mechanisms of patent system disclosure, protection, and subsidies with the background of China’s intellectual property system and enterprise management practices, and analyze the mechanism of technology strategy formation of three populations of subjects in the innovation ecosystem. Second, we introduced population evolution theory and the assumption of finite rationality to analyze the dynamic evolution of subject strategies in the innovation ecosystem, and propose that the technological strategies of subjects in the innovation ecosystem are the results of mutual learning and competition among enterprises under the assumption of finite rationality, which are consistent with the characteristics of ecological population and environment co-evolution. Third, we used two complementary research methods, dynamic evolutionary game and simulation, to study the game relationship between regulation strategy, protection strategy, and following strategy of innovation outcomes, which enriches the research on the evolutionary mechanism of technology strategy formation of innovation ecosystem subjects and deepens the theory related to technology competition.

This study establishes a tripartite revenue matrix of government and the leading and following firms using the evolutionary game theory model. The replicated dynamic equations of the three stakeholders are obtained, and the evolutionary stabilization strategies and corresponding conditions are calculated using the Jacobi matrix. Finally, numerical simulations are conducted to illustrate the impact of different trends of the relevant parameters on the evolutionary game of the three stakeholders according to the actual situation. The rest of the paper is organized as follows. Section 2 reviews and organizes the relevant literature in the context of government regulations, cooperative strategies, and evolutionary game theory. Section 3 describes the problem and parameters to model the evolutionary game for several topics and discusses some analytical results. In Section 4, simulation analysis is performed based on realistic situations. Section 5 presents the research conclusions, management insights, and practical implications of this paper.

## Literature review

This section presents a review of contemporary literature on the evolutionary game theory and government-firm relationships in innovation ecosystems and discusses gaps in the literature.

### Evolutionary game theory

Evolutionary dynamics suggest that in natural selection for survival of the fittest, cooperators are likely to be eliminated [20–23]. To study the evolution of cooperative group behavior, Nowak and May [24] introduced the evolutionary game theory to this complex network ecosystem. In the evolutionary game model, regardless of the respective stages of the participants and their initial strategies, they can change their strategies by observing, learning, and imitating other people’s behavior to adopt an evolved optimal stable strategy [25]. It describes the most important typical social interactions, such as conflicts or dilemmas, in quantitative terms [26]. According to evolutionary game theory, scholars have proposed the existence of multiple evolutionary mechanisms between groups, such as kin selection [27], direct reciprocity [28, 29], group selection [30], and indirect reciprocity [31]. Gradually, almost simultaneously with advances in network science, significant advances have been made in evolutionary game theory [22, 32–34], and it is no longer limited to the discipline of biology. This is mainly because of the interdisciplinary approach adopted in evolutionary game theory that links knowledge from biology, sociology, and economics, as well as mathematics, physics, and psychology [35–37].

The basic principles in evolutionary game theory have been widely applied to different fields such as species diversity [38], climate negotiations [39, 40], public health [41, 42], and traffic flow [43]. Duong and Pham [44] used evolutionary game theory to obtain asymptotic formulas for determining the continuous probability of a positive holomorphic line with a random polynomial, whose key ingredient was a degree index order bounded close to the random polynomial by an appropriate central smooth Gaussian process. Sekiguchi and Ohtsuki [45] described the stochastic evolution of an infinite population of 2 × 2 bimatrix game kinetics, obtaining a fixed probability of convergence of the evolutionary dynamics from a given primitive state to a specific absorbing state; they proved that evolutionary dynamics is biased toward fairness. Garay et al. [46] illustrated the role of time constraints in matrix games through the prisoner’s dilemma game, where additional time constraints ensured the existence of a single evolutionary stable strategy. Despite the aforementioned several practical branches and application areas, the fundamental problem that has been studied in the field of evolutionary game theory is the evolution of cooperation [47, 48]. Cooperation is an altruistic behavior that is costly but beneficial to others. Importantly, altruistic cooperation is the most important challenge to Darwinian evolution, and it forms the basis for understanding the major evolutionary transitions from single-celled organisms to complex animals and human societies [49, 50]. Thus, understanding the evolution of cooperation remains a challenge that has attracted research interest in both social and natural sciences.

### Innovation ecosystem government-business relationship

Due to the decentralized nature of expertise and the networked nature of technology development, firms cannot successfully conduct research and development (R&D) and innovation activities that only procure knowledge internally, leading to a paradigm shift from internal producer-led innovation to collaborative innovation and R&D [51–53]. This development is reflected in the concept of business ecosystems [54]; more recently, innovation ecosystems [8] have revealed how firms are becoming increasingly interdependent in their business and innovation activities. Value is often co-created within networks of firms, including collaboration and competition in different or even identical markets [55, 56]. To promote a mutually beneficial innovation ecosystem, especially to avoid unbalanced development between coastal and inland regions, the government can guide firms through policy support, such as procurement, price subsidies, financial support, and technology sharing models for collaborative innovation. The government can also rely on the establishment of information support and innovation exchange platforms or a service system centered on building an innovation service market to encourage enterprises for implementing technological complementary innovation [57]. Technological innovation in enterprises should adopt different cooperation models according to different types of innovations; for example, key technologies are suitable for independent innovation through internal R&D or cooperative innovation through technology alliances to obtain patents; however, other non-key technologies can obtain patent usage rights through market transactions [58].

Leading firms must choose innovation protection strategies that match their micro, meso, and macro environments according to their characteristics [59]. At the micro level, innovation protection strategy selection considers factors such as firm size, nature, resource capacity, innovation characteristics, and strategy matching [60–62]. Surveys by Yale, Carnegie Mellon, and other institutes indicate that technology secrets are more important than patents for both product and process innovations [63, 64]. Firms are more inclined to adopt patent protection for product innovations than for process innovations [65, 66]. At the meso level, leading firms are embedded in meso social networks, and innovation protection strategies are influenced by the meso social network environment [67, 68]. Industry technological complexity influences the choice of patents and technical secrets, and the role of patents is more prominent in pharmaceutical and chemical industries than in other industries [69]. Firms’ innovation protection strategies are also affected by factors such as supply and demand, the intensity of industry competition, number of competitors, threat of substitutes, industry structure, and industry life cycle [70, 71]. At the macro level, the innovation protection strategies of leading firms are governed by the institutional environment [72], which influences the innovation protection strategies of leading firms through three mechanisms, namely rational choice, homogeneous pressure, and organizational inertia [73]. Leading firms’ perceptions of institutions influence their judgment benchmarks and they rationally choose their innovation protection strategies under homogeneous pressures; moreover, their strategies remain stable over a range and historical period due to path-dependence effects [74, 75]. Due to the nature of easy knowledge spillover, the following firms in the innovation ecology in the later position can acquire spillover knowledge through patents, information interaction, personnel mobility, reverse engineering, and so on, and they learn to catch up with the leading firms [76]. Typically, following firms can adopt two strategies, namely imitation and substitution [17], which focus on copying the technological path of the leading firm by imitation and on the means for adopting different technological paths to achieve similar efficacy as the technological innovation of the leading firm, respectively. In an environment with a strong IPR regime, following firms prefer to adopt alternative strategies for avoiding losses from infringement lawsuits. Conversely, in a weak IPR protection environment, following firms prefer to adopt imitation strategies [77]. The strategy of leading firms to protect their IPRs also influences the strategy choice of following firms. When leading firms protect their innovations through patents and actively exercise their patent rights, following firms tend to adopt alternative strategies [13]. Additionally, perceptions of the executives in following firms are the key endogenous factors in their choice of following strategies [12]. Considering the game between leading and following firms, the choice of leading firms to protect innovations in the form of patents or technology secrets allows them to control the manner and intensity of information disclosure [78]. Following firms consider the value of the innovation, cost of imitation, and risk of infringement, and they chose whether to imitate. Based on the competitive dynamics resulting from the choices of leading and following firms, the optimal choice between competitors and imitators is evaluated by calculating returns based on the economic competition models (e.g., Gounod model) [79–81]. Mosel [82] found that considering the cost of patent protection, firms use technology secrets for smaller degrees of innovation and patent protection for larger degrees of innovation. Mihm et al. [83] used a simulation approach to study firms’ innovation protection strategy choice during the innovation race and pointed out that when two leading firms compete, the patenting strategy is better for both sides. When a leading firm competes with a following firm, it should adopt a technology secret strategy to protect innovation and a patent strategy for patent layout.

## Tripartite evolutionary game model

### Game players and behavioral strategies

Generally, inter-topic decision selection is studied using methods such as stochastic differential game [84] and integrated fuzzy decision approach [85]. Evolutionary games typically assume that participants are finitely rational and then study the dynamic evolutionary process of their strategies. The three-subject evolutionary game model is applied to the strategy choice between the government and the two types of firms [7]. Leading firms in core positions in the innovation ecosystem dominate the development of ecology and make important contributions to technological innovation, coordination of network relationships, knowledge externalities, and ecosystem evolution [9]. Following firms are in the non-core, subordinate, and supporting positions, resulting in ecological niche differences with leading firms through technological imitation, complementarity, and substitution, and promote the scale effect and synergy of the innovation ecosystem and systemic innovation upgrading [86]. However, core firm-led governance often lacks legal constraints, and because of the profit-seeking nature of firms, core firms often suffer from insufficient motivation or resource shortage when leading governance, which reflects that government market regulation is an effective policy tool for overcoming market failure and promoting sustainable economic growth [18]. Dynamic competitive partnerships among leading firms, following firms, and governments form the root of ecosystem variation, preservation, and sustainability [10]. The ecosystem view of innovation based on the ecology of differentiated populations provides a new perspective for explaining firms’ technological strategies. In this study, we analyzed the intrinsic mechanism of leading firms’ innovation protection strategies, following firms’ following strategies, and government regulation behavior in the self-organized evolution of an innovation ecosystem from the dynamic perspective of macro- and micro-interactions. Furthermore, we clarified the game relationship between multiple stakeholders in the innovation ecosystem and examined the impact of government regulation on firms’ innovation behaviors. The game relationships among multiple stakeholders in this study are as follows:

#### Local government strategy set for {market regulation, not market regulation}

First, the government requires a positive market competition environment regulation to increase the legitimacy of enterprises’ innovative behavior, as well as the probability of enterprises being punished for plagiarism and imitation, which enhances the risk of imitating innovation and pressurizes following enterprises. Second, local governments stimulate enterprises to innovate independently through measures such as tax incentives or price subsidies. Finally, local governments’ market regulation behavior gains policy benefits from the central government.

#### Leading corporate strategy set for {patent application, technology secrets}

As leading technology innovators, leading companies possess knowledge and technological advantages. To promote innovation ecology, positive externalities should be implemented through knowledge diffusion for diversification and scaling. Additionally, to maintain competitive advantage and leadership in the ecosystem, the rhythm and speed of knowledge spillover should also be considered [87]. Therefore, leading firms choose to protect innovations via patents or technology secrets, which enable them to control the manner and intensity of information disclosure [78]. Patents are disclosed in exchange for protection. It allows leading firms to promote the diffusion of explicit knowledge and master the speed of diffusion and the targets of diffusion through institutional means, which help the leading firms in grasping the voice and initiative of the innovation ecology. However, the disadvantages of patents is that their monitoring and governance are challenging, and the protection of their effectiveness necessitates reliance on institutions. Technology secrets are advantageous as they help protect tactical knowledge and maintain exclusivity of new technology; however, they are associated with a risk of spillover, which makes it challenging to maintain rights.

### Corporate strategy set for {limitation, substitution}

Follower firms are key drivers of development in the innovation ecosystem. Although they are slightly subordinate to leading firms, their bargaining power may improve, and they may even become new leader firms. Follower firms choose whether to imitate a leading firm, considering the value of innovation, cost of imitation, and risk of infringement. The imitation strategy is less costly and difficult but is easily constrained by the system. Conversely, the substitution strategy is costly and difficult, but it presents no risk of infringement; moreover, it offers an opportunity to the firms to change their position and bargaining power [88].

**Fig 1:**
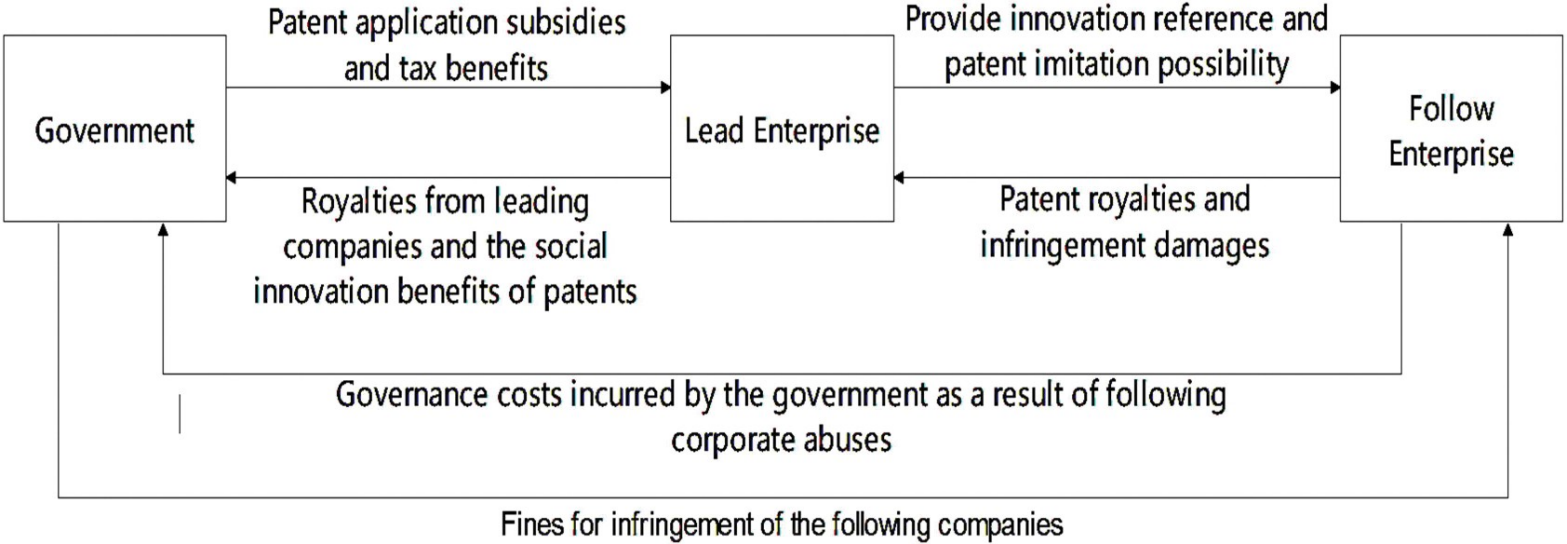
Game relationship among three subjects.

Herein, the three participants of the evolutionary game are the government, leading firm, and following firm. The government, as the regulator, has two regulatory strategy choices: market regulation (G1) and non-market regulation (G2). The behavioral strategy of leading firms is divided into two options, patenting (L1) and technology secrecy (L2). Following firms have two strategic tactics, imitation (F1) and substitution (F2). Based on the above description, the intertwined game relationship of the three parties is illustrated in **Fig 1**.

### Basic assumptions and model parameters

Based on the analysis of the dynamic game relationship between the three stakeholders, we propose the following hypotheses:

Hypothesis 1: The innovation ecosystem has a population of leading firms comprising m leading firms li (i = 1, 2, 3,…, m) and a population of following firms comprising n following firms fj (j = 1, 2, 3,…, n).

Hypothesis 2: Innovation ecosystem evolution is a process of dynamic adjustment of strategies of two populations under a certain institutional environment. The strategy changes of individuals in the populations are in accordance with the replication dynamic equation of biological evolution, and firms compete while learning from each other and can judge and change their strategies. According to their learning object gains, individual strategies are continuously adjusted, and the game cycle is the product life cycle.

Hypothesis 3: All three participants in the game are finite rational, and the probability that the local government chooses market regulation is x, 0 ≤ x ≤ 1; the probability that the leading firm chooses to apply for a patent is y, 0 ≤ y ≤ 1; and the probability that the following firm chooses to imitate is z, 0 ≤ z ≤ 1.

Based on these assumptions, the evolutionary game payoff function is constructed. Technological innovations of leading firms lead to cost leadership or differentiation advantages, thereby leading to the evolution of the innovation ecosystem. Following firms catch up with the technological innovation of leading firms and can produce homogeneous products; under a certain environment of IPR system under government regulation, leading firms that adopt patent strategies can obtain license fees. Leading firms can obtain other benefits such as litigation damages through patent licensing and litigation, and they can incur transaction costs due to contracts, negotiations, and information search, while reducing the difficulty of imitation and substitution for following firms [89]. Leading firms that adopt technology secret strategies can suppress knowledge spillover and increase the difficulty of imitation and substitution; however, they are at a risk of losing exclusivity. Moreover, leading firms receive first-mover advantages such as time, brand, and channels due to the earlier entry of their product in the market. Therefore, it is assumed that in one game cycle, leading firms gain R and following firms gain δR, with 1> δ > 0. Accordingly, Table 1 further describes the relevant notations and definitions.

**Table 1:** Description of parameter symbols.

Parameters	Description
R	Initial gains for leading companies
δR	Follow the company’s initial earnings
θR	Follow business infringement fees or patent license fees
S	Government subsidies for patent applications by leading companies
C1	R&D costs for leading companies
C2	Costs of filing and maintaining patents for leading companies
C3	Government Regulatory Costs
R1	Social innovation benefits of patents from leading companies
W	Follow the fines paid to the government when companies infringe
Α	Loss factor due to imitation difficulty
Μ	No patent disclosure is increased learning (including imitation and substitution) difficulty factor
Β	Loss factor due to substitution difficulty
θ	Patent protection intensity of leading companies

### Evolutionary game analysis of replicated dynamic equations

In this study, we used replicated dynamic equations to portray the dynamic evolution of the strategies of the three parties, aiming to clarify the mechanism of local government market regulation on the innovation behavior of leading and following firms. According to the model assumptions, the payoff matrix of the three-party game was obtained and is presented in Tables 2 and 3.

**Table 2:** Game matrix of each subject under government regulation (x).

**x**		**Z**		
		Government	Leading company	Following company
	Y	-S+W-C3+R1	R-C1-C2+S+θR	δR-θR-W-αC1
	1-y	-C3	R-C1	δR-μαC1
		**1-z**		
		Government	Leading company	Following company
	Y	-C3+R1-S	R+S-C1-C2	δR-βC1
	1-y	-C3	R-C1	δR-μβC1

**Table 3:** Game matrix of each subject under government non-regulation (1-x).

**1-x**	**Z**			
		Government	Leading company	Following company
	y	R1	R+θR-C1-C2	δR-θR-αC1
	1-y	0	R-C1	δR-μαC1
	**1-z**			
		Government	Leading company	Following company
	y	R1	R-C1-C2	δR-βC1
	1-y	0	R-C1	δR-μβC1

Following constraints are considered: ① C1>C2, R&D cost > patent cost; ② μ>1, adoption of technology secrets by leading enterprises increases the learning difficulty and cost of following enterprises.

Based on the above analysis and the game matrix, the expectations of the government and leading and following firms were calculated to derive the replication dynamics equation. For brevity, only the expectations of the government and the replication dynamic equation are presented in this study. rG1 denotes the revenue when the government regulates, and pG, pL, and pF denote the probability of the corresponding decisions of the government, leading firm, and following firm, respectively.

First, we calculated the corresponding expectations, E(G1) and E(G2), under the conditions when the government regulates and when the government does not regulate, and then, multiplied them with the probability of the corresponding decision to obtain the government’s benefit expectation, E(G), as given in Eqs (1)–(3).


$$\begin{array}{l} E(G1)=\sum rG1*pL*pF\\ \;=(R1+W-S-C3)\mathrm{yz}+(‐C3+R1-S)y(1‐z)\\ \;‐C3(1‐y)z‐C3(1‐y)(1‐z)\\ \;=R1y‐C3‐\mathrm{Sy}+\mathrm{Wyz} \end{array}$$ (1)

$$\begin{array}{l} E(G2)=\sum rG2*pL*pF\\ \;=R1\mathrm{yz}+R1y(1‐z)\\ \;=R1y \end{array}$$ (2)

$$\begin{array}{l} E(G)=pG1*E(G1)+pG2*E(G2)\\ \;=x*[\mathrm{R1y}‐C3‐\mathrm{Sy}+\mathrm{Wyz}]+(1‐x)R1y\\ \;=\mathrm{R1y}‐C3x‐S\mathrm{xy}+\mathrm{Wxyz} \end{array}$$ (3)

Finally, the replication dynamic equation F(x) for the government decision was calculated based on the benefit expectation, as given in Eq (4).


$$\begin{array}{l} F(x)=\frac{\mathrm{dx}}{\mathrm{dt}}\\ \;=x[E(G1)‐E(G)]\\ \;=x(1‐x)[E(G1)‐E(G2)]\\ \;=x(x‐1)(C3+\mathrm{Sy}‐\mathrm{Wyz}) \end{array}$$ (4)

The detailed process of calculating the government earnings expectation and replication dynamic equation is presented above. Similarly, the earnings expectations, E(L) and E(F), and the replication dynamic equation, F(y) and F(z), are calculated for the leading and following firms, as given in Eqs (5)–(8).


E(L)=R‐C1‐C2y+Sxy+Rθyz (5)

$$\begin{array}{l} E(F)=‐z(C1\mu \alpha ‐\partial R+C1\alpha y+R\theta y+\mathrm{Wxy}‐C1\mu \alpha y)\\ \;‐(z‐1)(\partial R‐C1\mu \beta ‐C1\beta y+C1\mu \beta y) \end{array}$$ (6)

F(y)=‐y(y‐1)(Sx‐C2+Rθz) (7)

F(z)=z(z‐1)(C1μα‐C1μβ+C1αy‐C1βy+Rθy+Wxy‐C1μαy+C1μβy) (8)

### Stability analysis of agent evolution game

In this study, the stability of the equilibrium point in the evolutionary game model was obtained by analyzing the Jacobi matrix. Eq (9) is the Jacobi matrix formula, and Eq (10) is the matrix obtained by replicating the dynamic equations according to each subject considered in this study.


$$J=[\begin{array}{lll} \frac{\partial F(x)}{\partial x} & \frac{\partial F(x)}{\partial y} & \frac{\partial F(x)}{\partial z}\\ \frac{\partial F(y)}{\partial x} & \frac{\partial F(y)}{\partial y} & \frac{\partial F(y)}{\partial z}\\ \frac{\partial F(z)}{\partial x} & \frac{\partial F(z)}{\partial y} & \frac{\partial F(z)}{\partial z} \end{array}]$$ (9)

$$J=[\begin{array}{lll} \left(2x‐1)(C3+\mathrm{Sy}‐\mathrm{Wyz}\right) & x(x‐1)(\mathrm{S-Wz}) & ‐\mathrm{Wxy}(x‐1)\\ ‐\mathrm{Sy}(y‐1) & ‐(\mathrm{2y-1})(\mathrm{Sx-C2+R}\theta z) & \mathrm{-R}\theta y(y‐1)\\ \mathrm{Wyz}(\mathrm{z-1}) & z(\mathrm{z-1})(\mathrm{C1}\alpha \mathrm{-C1}\beta \mathrm{+R}\theta +W\mathrm{x-C1}\mu \alpha \mathrm{+C1}\mu \beta ) & \left(\mathrm{2z-1})(\mathrm{C1}\mu \alpha \mathrm{-C1}\mu \beta \mathrm{+C1}\alpha y‐C1\beta y+R\theta y+\mathrm{Wxy}‐C1\mu \alpha y+C1\mu \beta y\right) \end{array}]$$ (10)

Assuming F (x) = 0, F (y) = 0, and F (z) = 0, we obtained 14 equilibrium points for all equilibria of this system. Of these, eight were pure strategy solutions and the remaining were non-pure strategy solutions. The equilibrium solution of the three-way evolutionary game is a strict Nash equilibrium; therefore, only the asymptotic stability of only eight points is discussed herein. According to the Liapunov discriminant [90], when all the eigenvalues are negative, the equilibrium point corresponding to the Jacobi matrix is the evolutionary stability point of the system of differential equations; otherwise, when the eigenvalues are 0, it is an unstable point, that is, a saddle point. System stability was analyzed by solving the eigenvalues of the Jacobi matrix corresponding to each equilibrium point (Table 4).

**Fig 2:**
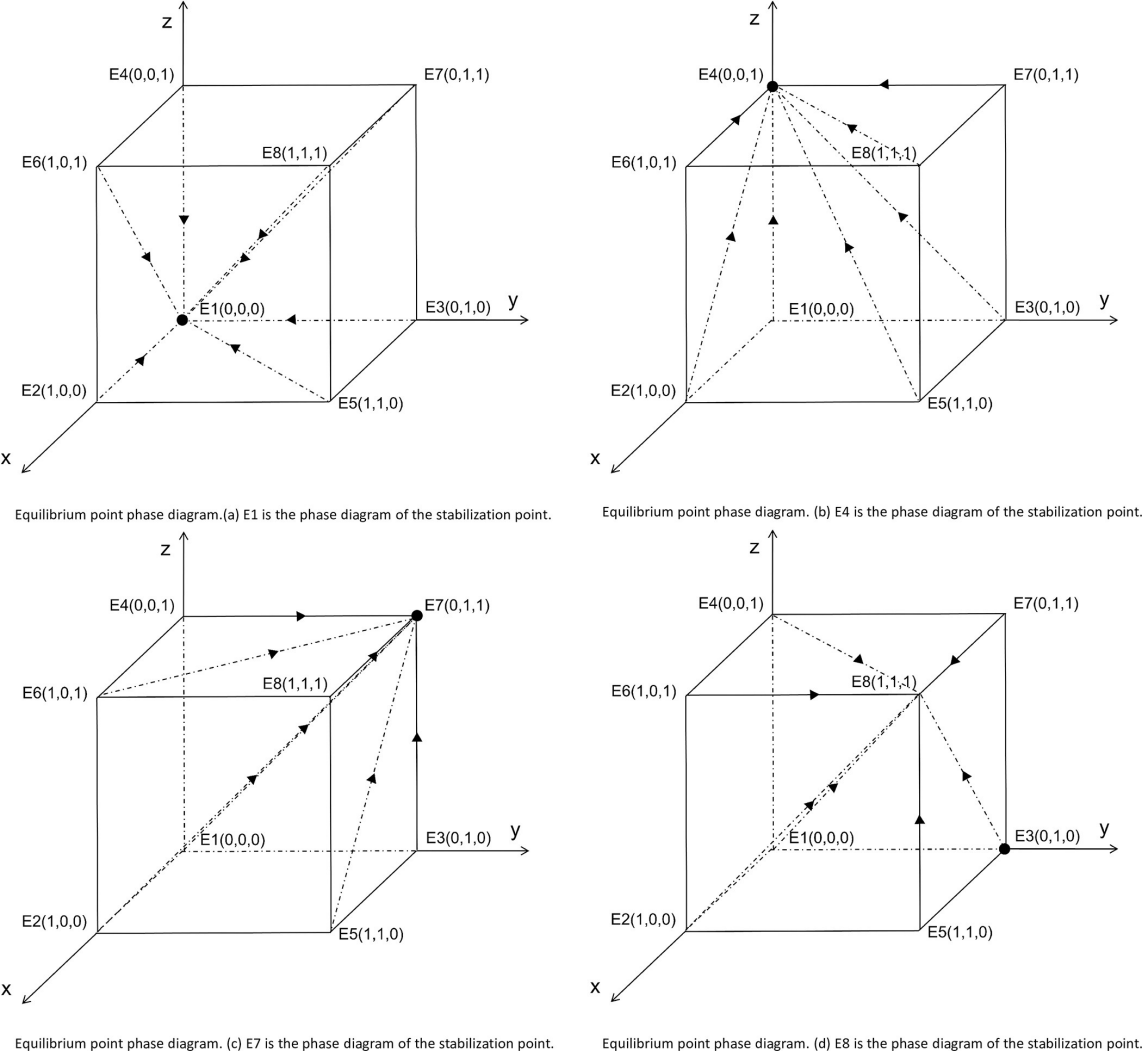
Equilibrium point phase diagram. (a) E1 is the phase diagram of the stabilization point. (b) E4 is the phase diagram of the stabilization point. (c) E7 is the phase diagram of the stabilization point. (d) E8 is the phase diagram of the stabilization point.

**Table 4:** Eigenvalues and stability points of Jacobi matrix.

Point	λ1	λ2	λ3	Stability
**E1(0,0,0)**	C1βμ-C1αμ	-C2	-C3	The equilibrium point is stable when Rθ < C2 and β < α. (Fig 2(A) for the phase diagram)
**E2(1,0,0)**	C3	C1βμ-C1αμ	S-C2	Instability point
**E3(0,1,0)**	C2	- C3-S	C1β—C1α—Rθ	Instability point
**E4(0,0,1)**	Rθ-C2	C1αμ-C1βμ	-C3	The equilibrium point is stable when Rθ < C2 and α < β. (Fig 2(B) for the phase diagram)
**E5(1,1,0)**	C2-S	C3+S	C1β—C1α—W–Rθ	Instability point
**E6(1,0,1)**	C3	C1αμ-C1βμ	S-C2+Rθ	Instability point
**E7(0,1,1)**	C2-Rθ	W-C3-S	C1α-C1β+Rθ	The equilibrium point is stable when C2<Rθ<C1β-C1α and W<C3+S. (Fig 2(C) for the phase diagram)
**E8(1,1,1)**	C2-S-Rθ	C3+S-W	W+C1α-C1β+Rθ	The equilibrium point is stable when C2<Rθ<C1β-C1α and C3+S<W. (Fig 2(D) for the phase diagram)

### Numerical simulation of the tripartite evolutionary game

Stable strategies for replicating dynamic equations in evolutionary games provide ideas for understanding the dynamic evolution of technological strategies of fully rational subjects in the leading and following firm populations in a differentiated environment. Agent-based modeling and simulation (ABMS) is a bottom-up modeling approach that describes complex self-organization phenomena and law emergence by portraying the roles of individuals, inter-individual interactions, and environmental selection [91]. The multi-subject simulation method can define the interaction game and learning mode between the leading and following firms, simulate the process of their strategy evolution, analyze and reveal the formation and evolution mechanism of firms’ innovation protection strategies, and present the following strategy under the assumption of finite rationality [92, 93]. In contrast, subjects are finite and rational, and incomplete information is available in the environment. To further investigate the evolutionary mechanism of the system based on the replication of constraints and kinetic equations, the evolutionary process was simulated using MATLAB software in this study. Parameter assignment was used to verify the stable equilibrium point of the evolution of the three-party strategy under different conditions and the influence of each key factor on the evolutionary equilibrium of each subject’s strategy and further verify the existence of the stable point of the Jacobi matrix in Section 3.3. Referring to Manman Wang et al. [7], to satisfy the model requirements, values are assigned to variables based on the following principles: first, considering the actual situation, such as the relevant provisions of patent law, which stipulate that enterprises intentionally infringing patent rights shall be fined 1–5 times of the direct loss incurred; second, measuring government subsidies based on the joint influence of government subsidy policy and tax relief policy on patents; third, based on the principle of equilibrium of equations, the initial parameters are assigned. In this section, we discuss the main results and provide explanations and some additional clarifications about these results.

### Equilibrium point simulation analysis

#### Scenario 1

**Fig 3:**
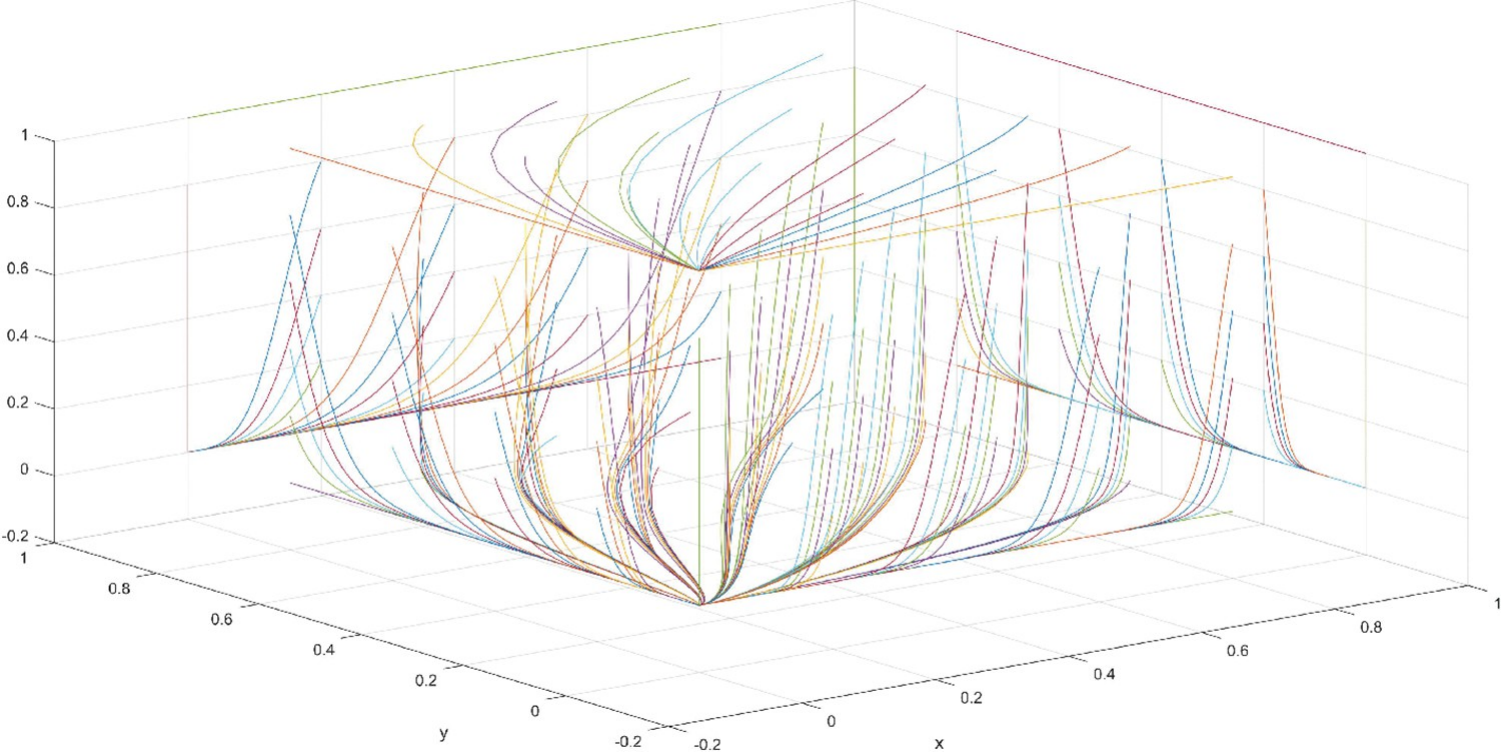
Three-dimensional view of Scenario 1 three-way evolutionary game diagram.

**Fig 4:**
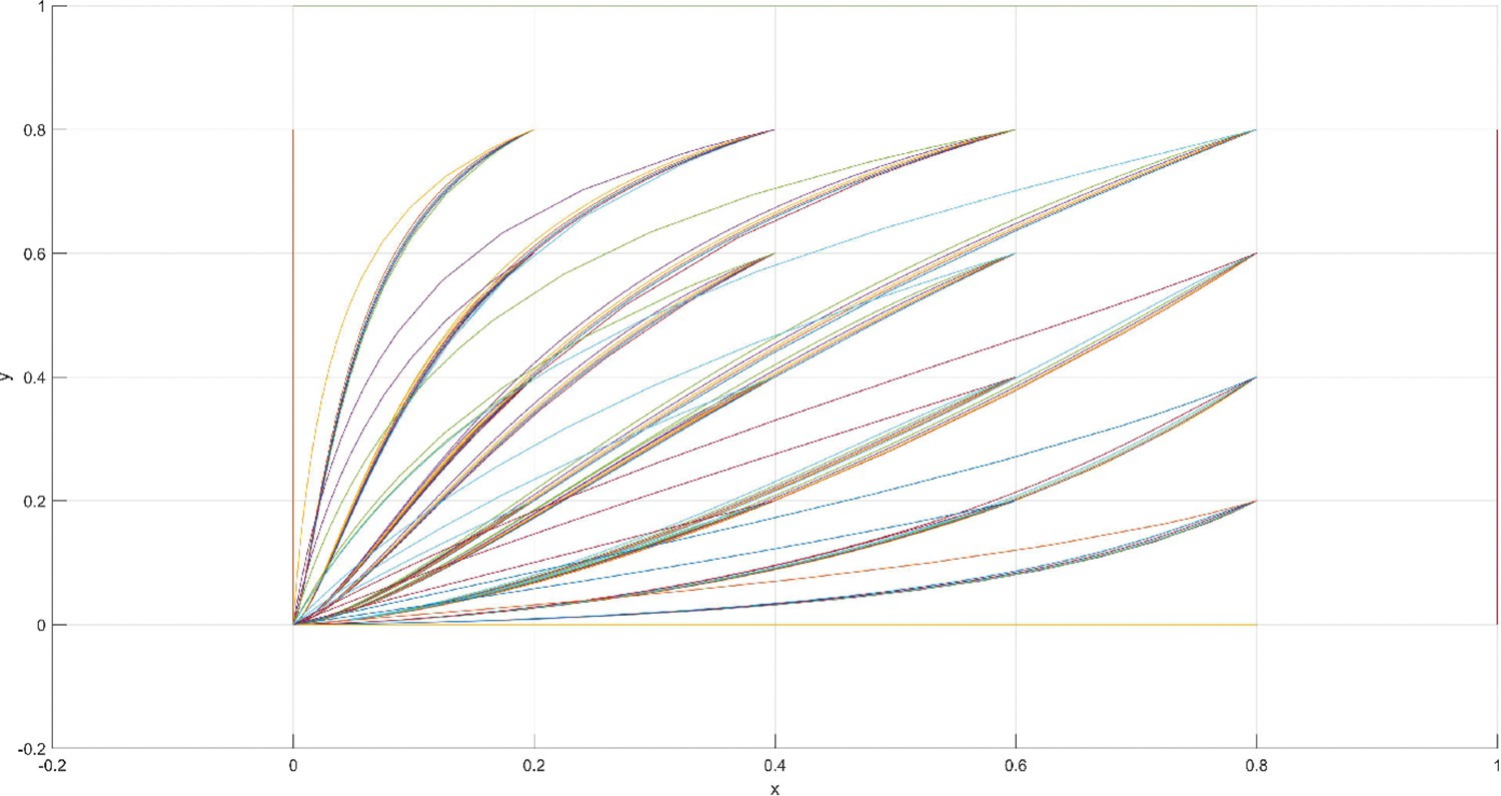
Scenario 1 x-y plane three-way evolutionary game diagram.

**Fig 5:**
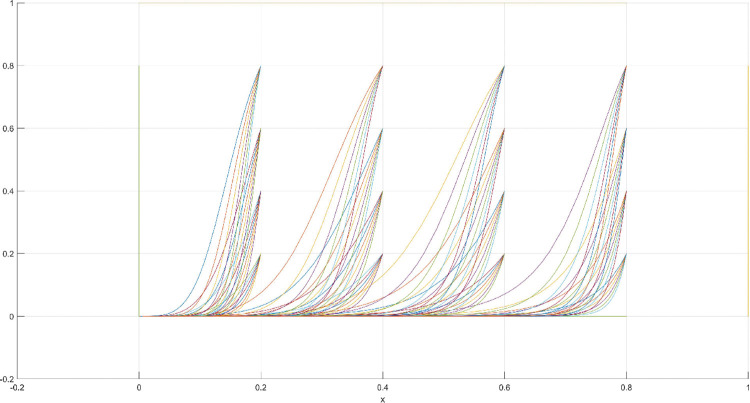
Scenario 1 x-z plane three-way evolutionary game diagram.

**Fig 6:**
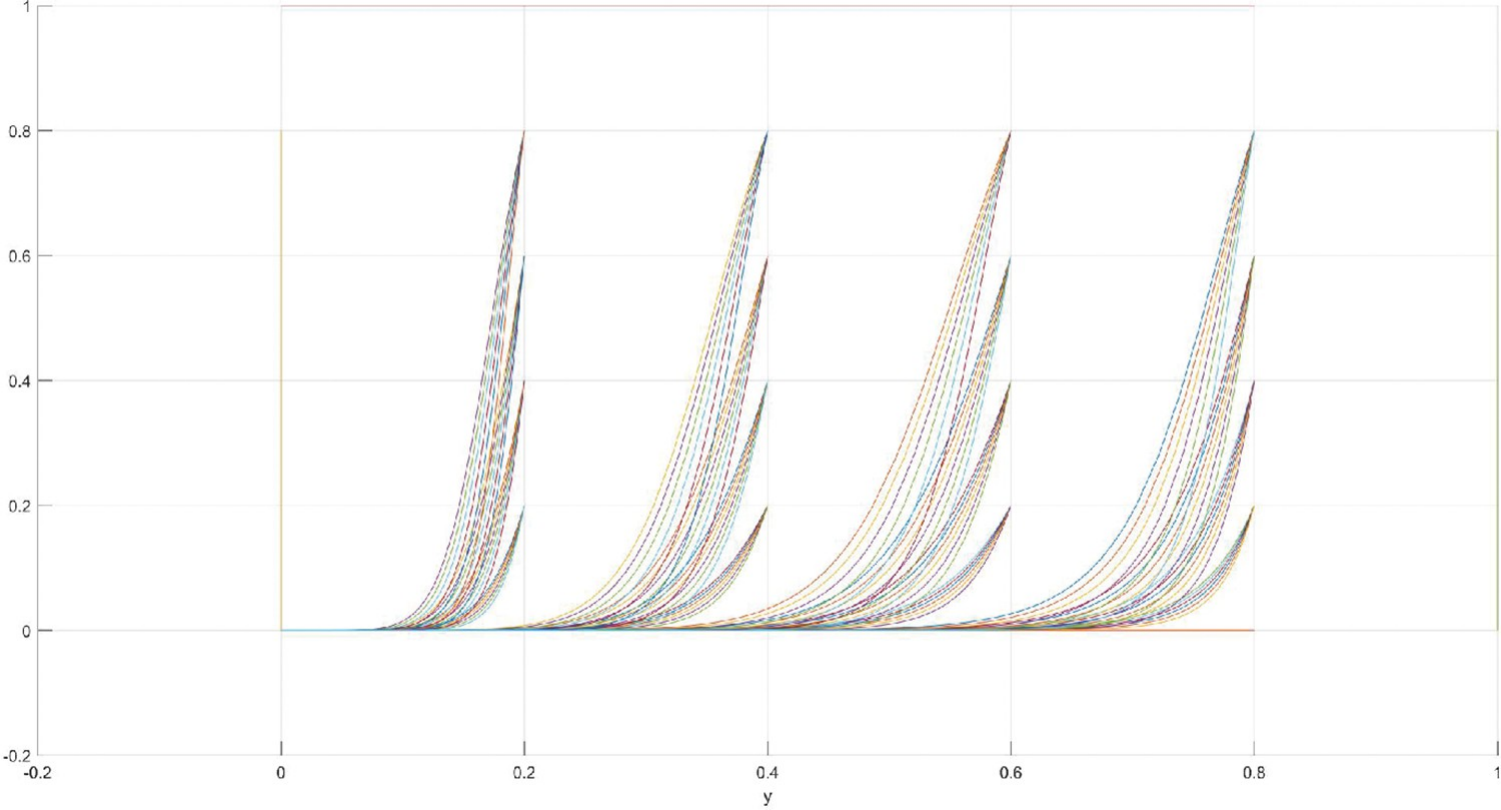
Scenario 1 y-z plane three-way evolutionary game diagram.

The conditions of Scenario 1 are that the patent infringement compensation obtained by the leading company is less than the cost of patent application and maintenance; the difficulty of replacing the innovation of the leading company by the following company is less than the difficulty of imitation, implying that the cost of replacement is less than the cost of imitation. At this point, following enterprises must choose to replace the innovation of leading enterprises with their own innovation because it involves the lowest innovation cost and no risk of infringement. In this case, if leading enterprises choose to apply for a patent instead, they need to pay the patent fee, thereby increasing the cost; leading enterprises choose to use the innovation as a secret technology, that is, differentiation strategy, to maintain competitive advantage in the market. At this time, the market does not exhibit infringement and other behaviors, and leading firms choose to work in line with government regulation. According to the aforementioned analysis, the values of parameters in line with Scenario 1 are assumed as follows: R = 100, S = 10, C1 = 50, C2 = 30, C3 = 20, R1 = 200, W = 5, δ = 0.5, α = 4, β = 2, μ = 2, and θ = 0.1; these values are substituted into the replication dynamic equation, and the evolutionary game diagram was drawn (Figs 3–6). x, y, and z tended to be at the far point E1 (0,0,0) with time, which represents the stable equilibrium point of the evolutionary game, and the corresponding strategy combination is {government non-regulation, technology secret, substitution}, which is consistent with the result of the stable point analysis of the Jacobi matrix in Section 3.3.

#### Scenario 2

**Fig 7:**
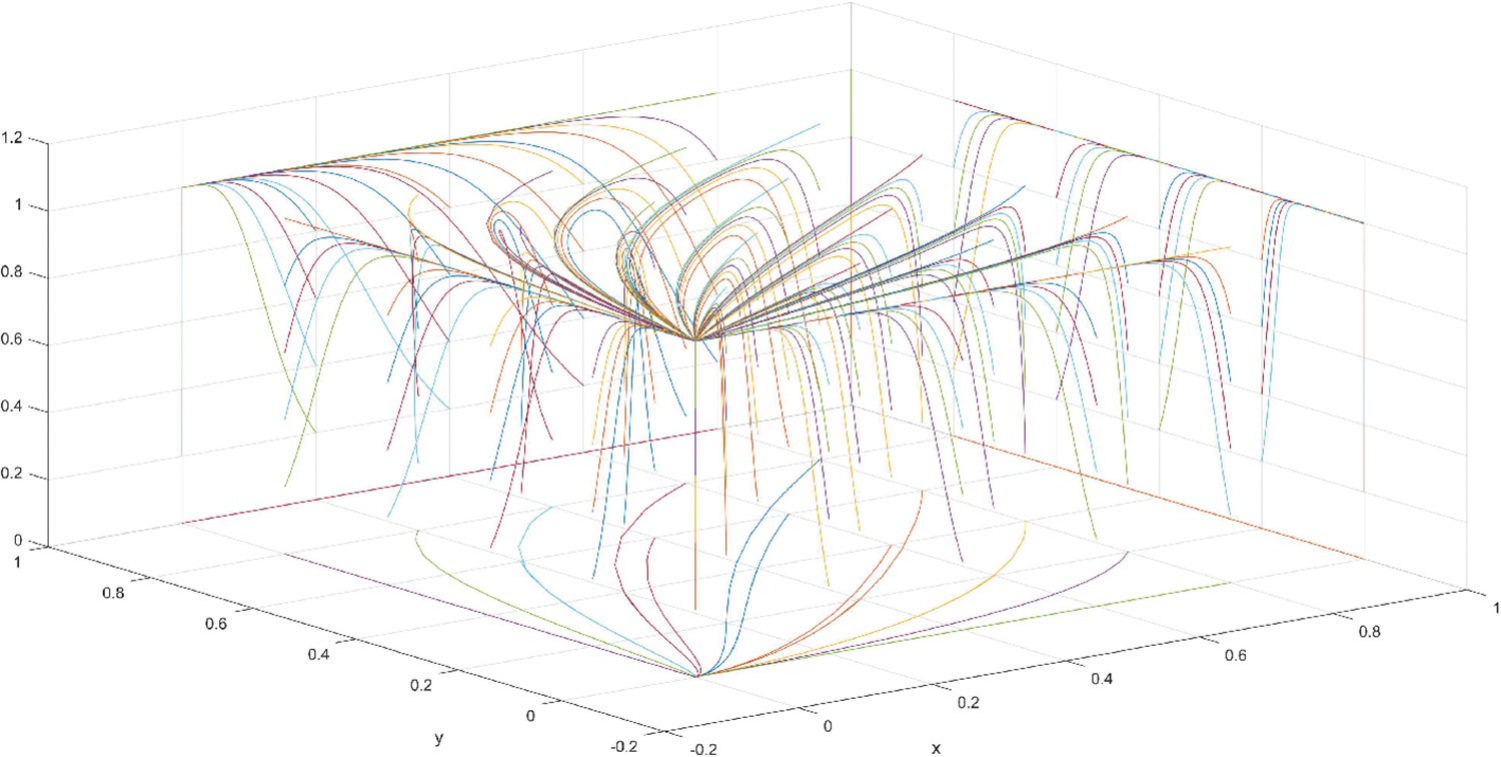
Three-dimensional view of Scenario 2 three-way evolutionary game diagram.

**Fig 8:**
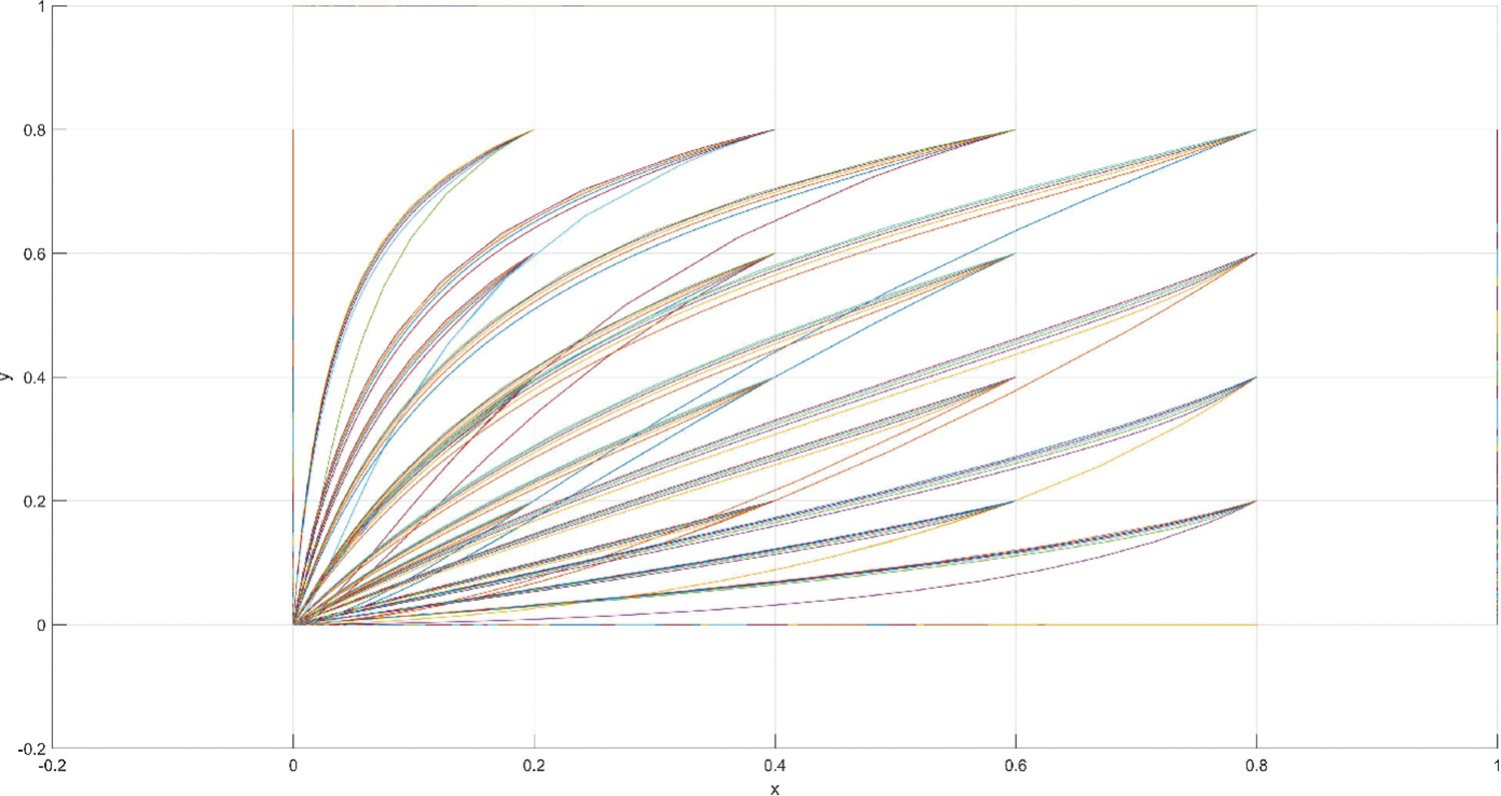
Scenario 2 x-y plane three-way evolutionary game diagram.

**Fig 9:**
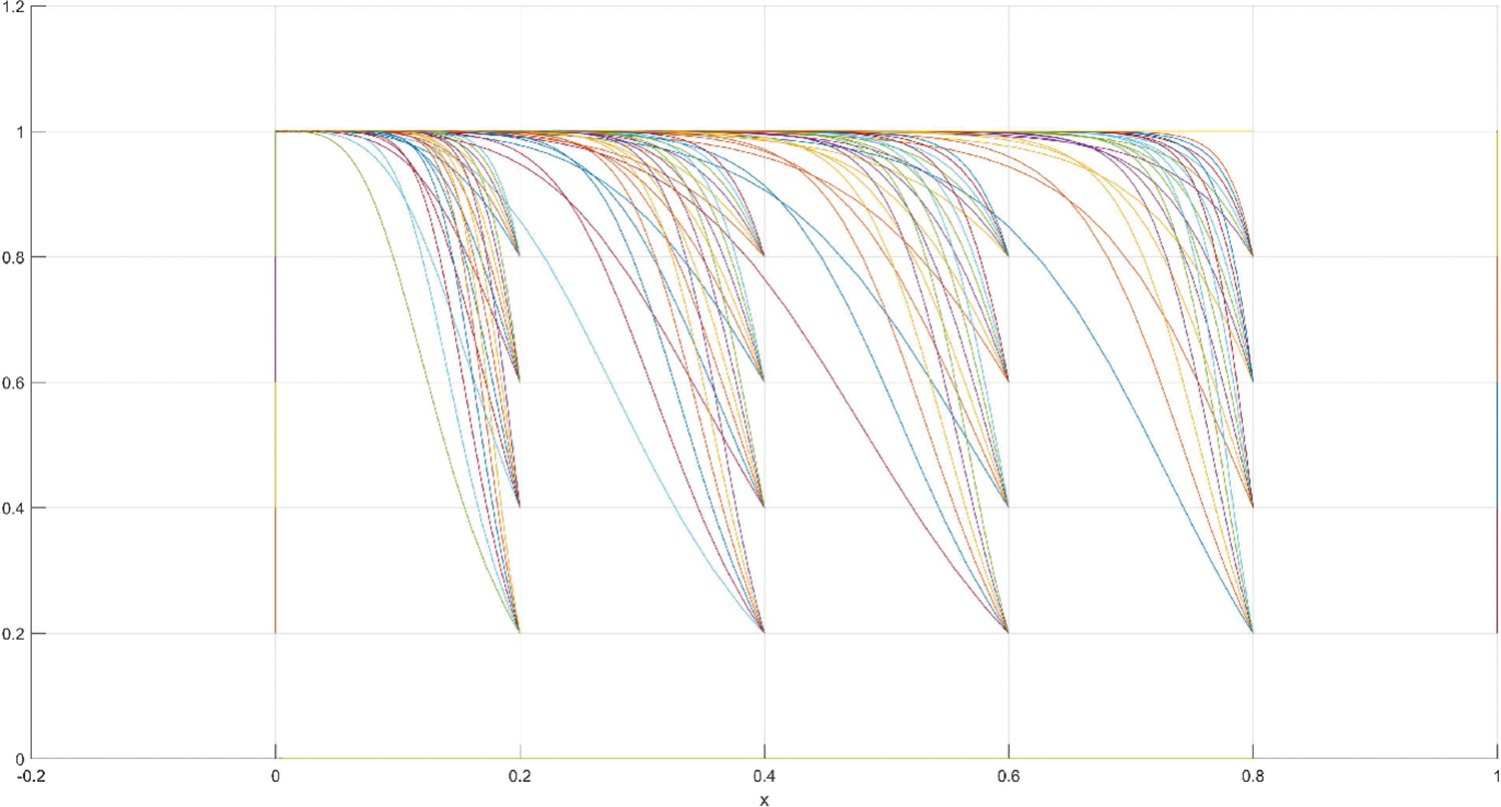
Scenario 2 x-z plane three-way evolutionary game diagram.

**Fig 10:**
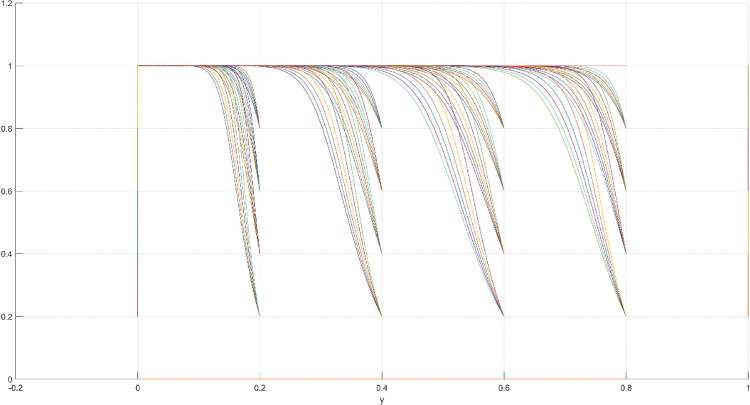
Scenario 2 y-z plane three-way evolutionary game diagram.

The condition of Scenario 2 is that the patent royalty or infringement compensation cost accessible to leading firms is less than the cost of patent application and protection; the difficulty of replacing the innovation of leading firms by following firms is greater than the difficulty of imitation, that is, the cost of replacement is greater than the cost of imitation. At this time, leading enterprises will not spend energy to apply for or maintain the innovation patent because they will be at a loss. For the followers, the enterprises’ decision depends on the difficulty of imitation and substitution of the innovation of the leading enterprise. At this point, if the cost of imitating the innovation of following firms is lower than the cost of replacing the innovation, then following firms will choose to imitate. Then, promoting the effectiveness of innovation in the whole market without disclosing their innovation results is difficult for leading firms. The government does not regulate in order to reduce the cost to a minimum. According to the above analysis, the parameter values conforming to Scenario 2 are assumed as follows: R = 100, S = 10, C1 = 50, C2 = 30, C3 = 20, R1 = 200, W = 5, δ = 0.5, α = 2, β = 4, μ = 2, and θ = 0.1; these values are substituted into the replication dynamic equation, and the evolutionary game diagram was drawn (Figs 7–10). At this point, x, y, and z converge to point E4 (0,0,1) with time, which is consistent with the equilibrium point of the evolutionary game in the Jacobi matrix, corresponding to the measurement of the slight combination of {government non-regulation, technology secrets, imitation}.

#### Scenario 3

**Fig 11:**
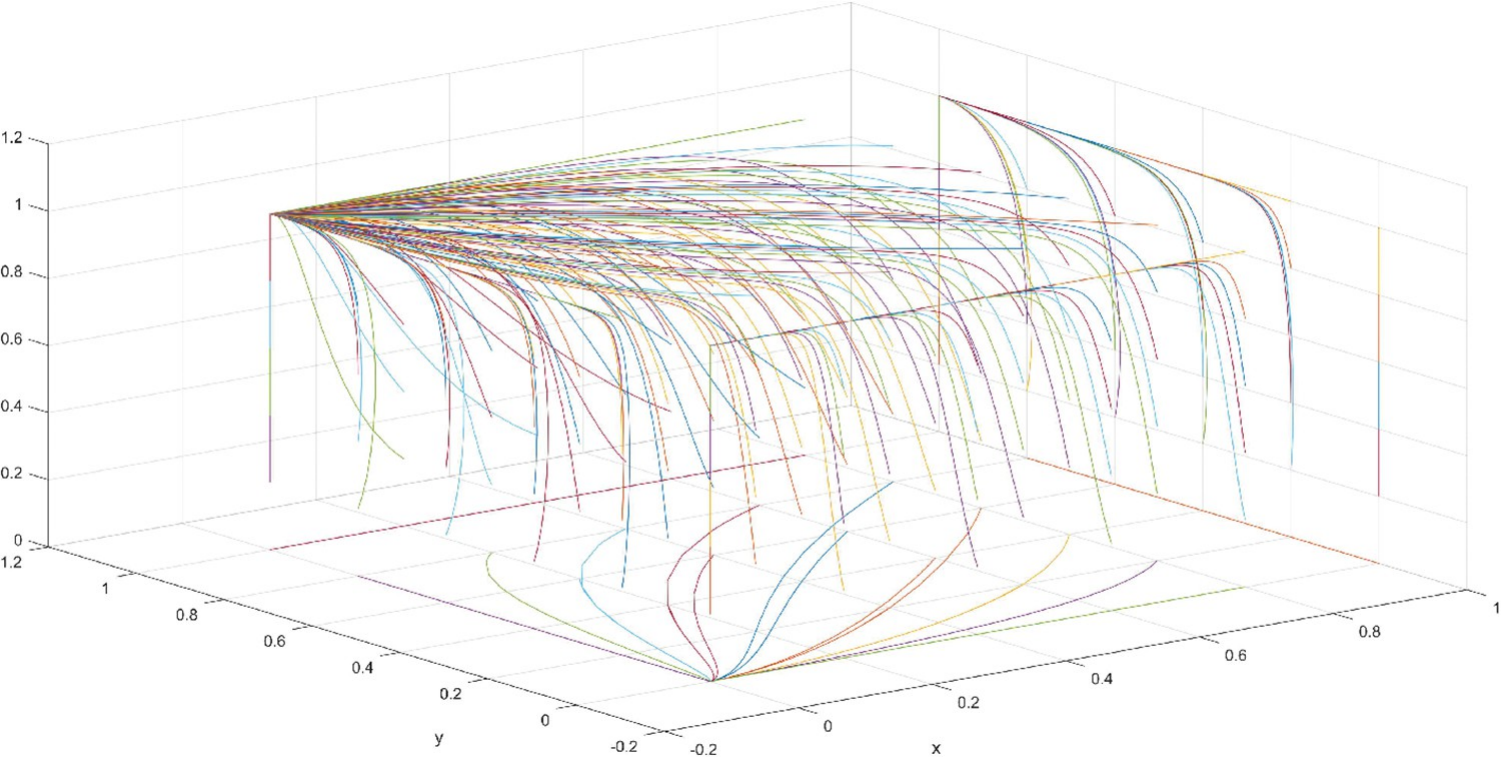
Three-dimensional view of Scenario 3 three-way evolutionary game diagram.

**Fig 12:**
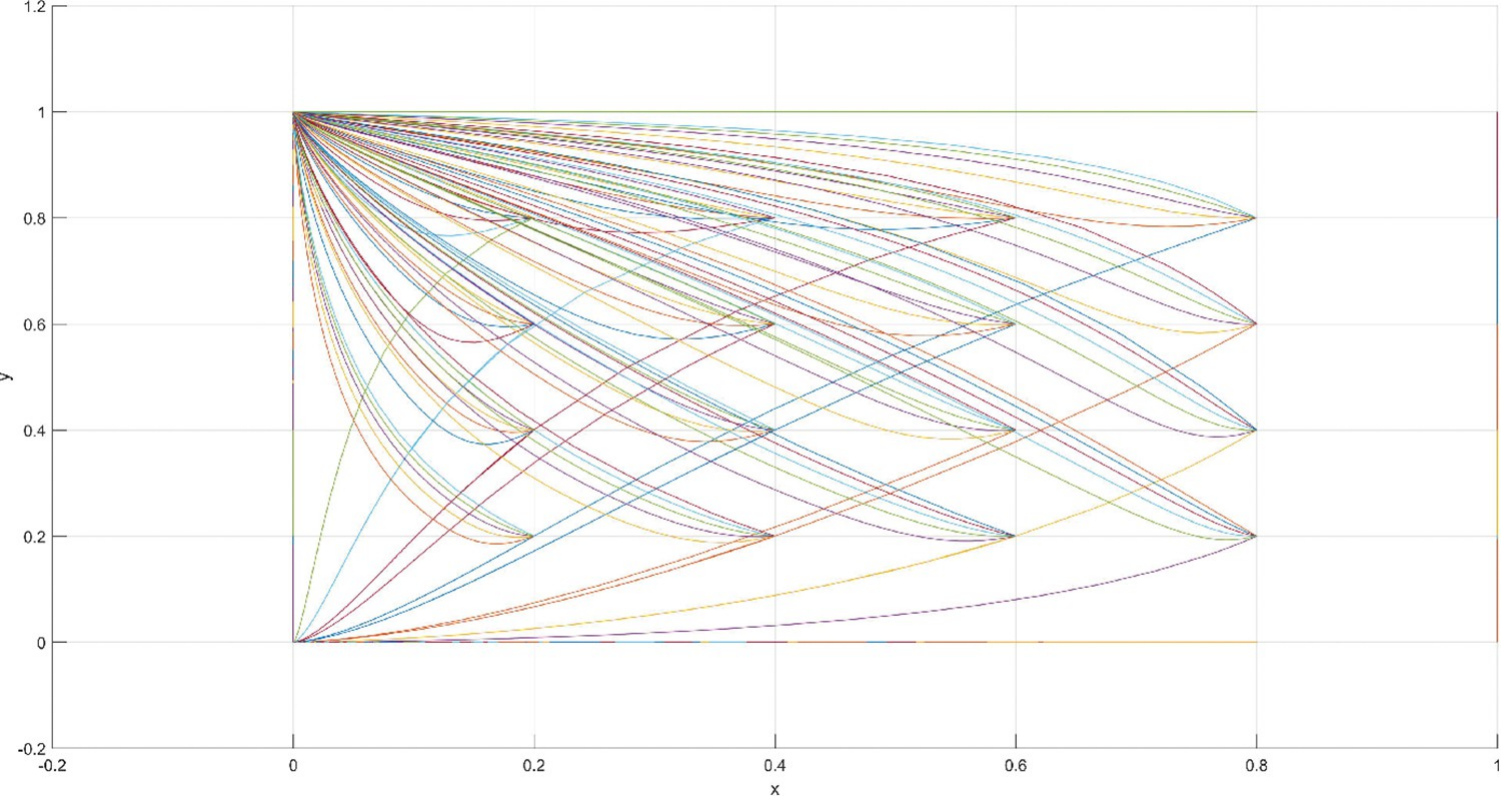
Scenario 3 x-y plane three-way evolutionary game diagram.

**Fig 13:**
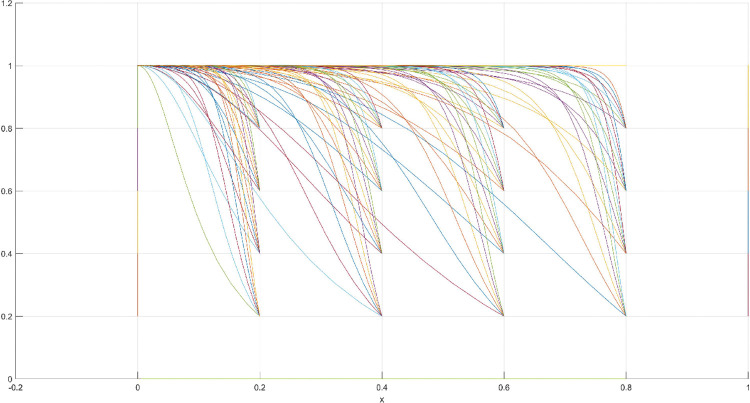
Scenario 3 x-z plane three-way evolutionary game diagram.

**Fig 14:**
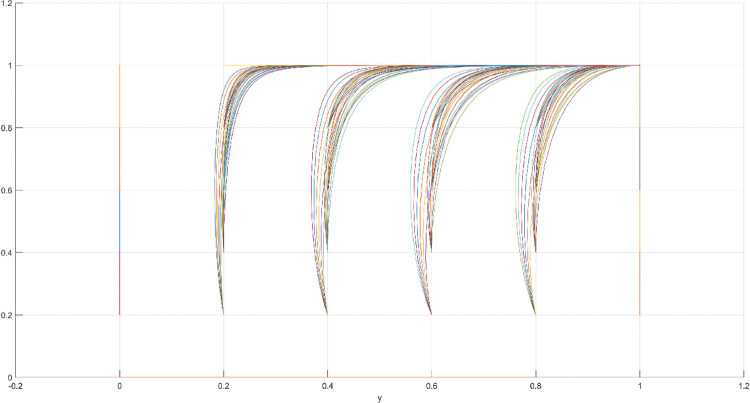
Scenario 3 y-z plane three-way evolutionary game diagram.

Scenario 3 is based on the conditions that the leading firms’ cost of applying for and maintaining a patent is less than the royalty or compensation received, and the government’s revenue from fines is less than the sum of regulatory costs and patent subsidies. In this case, leading firms will choose to apply for a patent. Then, the publicly available patented technology leads to further development in the innovation market, generating benefits for the government. If the innovation is extremely difficult to replace for following firms, and the replacement cost is higher than the imitation cost in addition to the royalty payment to leading firms, then following firms choose the imitation strategy to reduce costs. Moreover, if the fine that the government can collect when regulating is less than the cost of patent subsidy and regulation, then the government chooses not to regulate. Based on the aforementioned analysis, the assumed parameters of Scenario 3 are as follows: R = 100, S = 10, C1 = 50, C2 = 30, C3 = 20, R1 = 200, W = 5, δ = 0.5, α = 2, β = 4, μ = 2, and θ = 0.5. These values are substituted into the replication dynamic equation, and the evolutionary game diagram was drawn (Figs 11–14). x, y, and z evolve over time toward point E7 (0,1,1), which is consistent with the equilibrium point of the evolutionary game in the Jacobi matrix, and the corresponding strategy combination is {government non-regulation, patent application, imitation}.

#### Scenario 4

**Fig 15:**
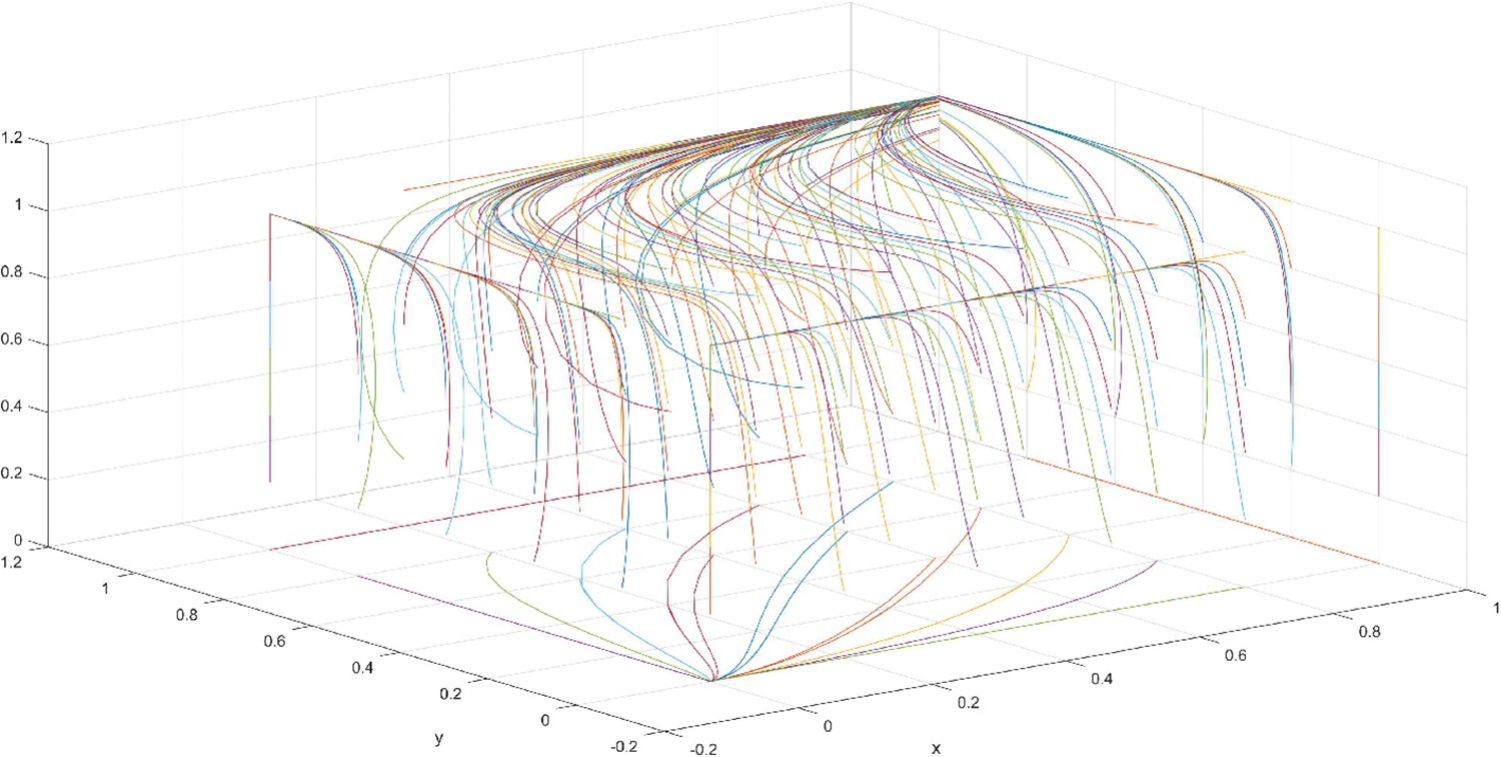
Three-dimensional view of Scenario 4 three-way evolutionary game diagram.

**Fig 16:**
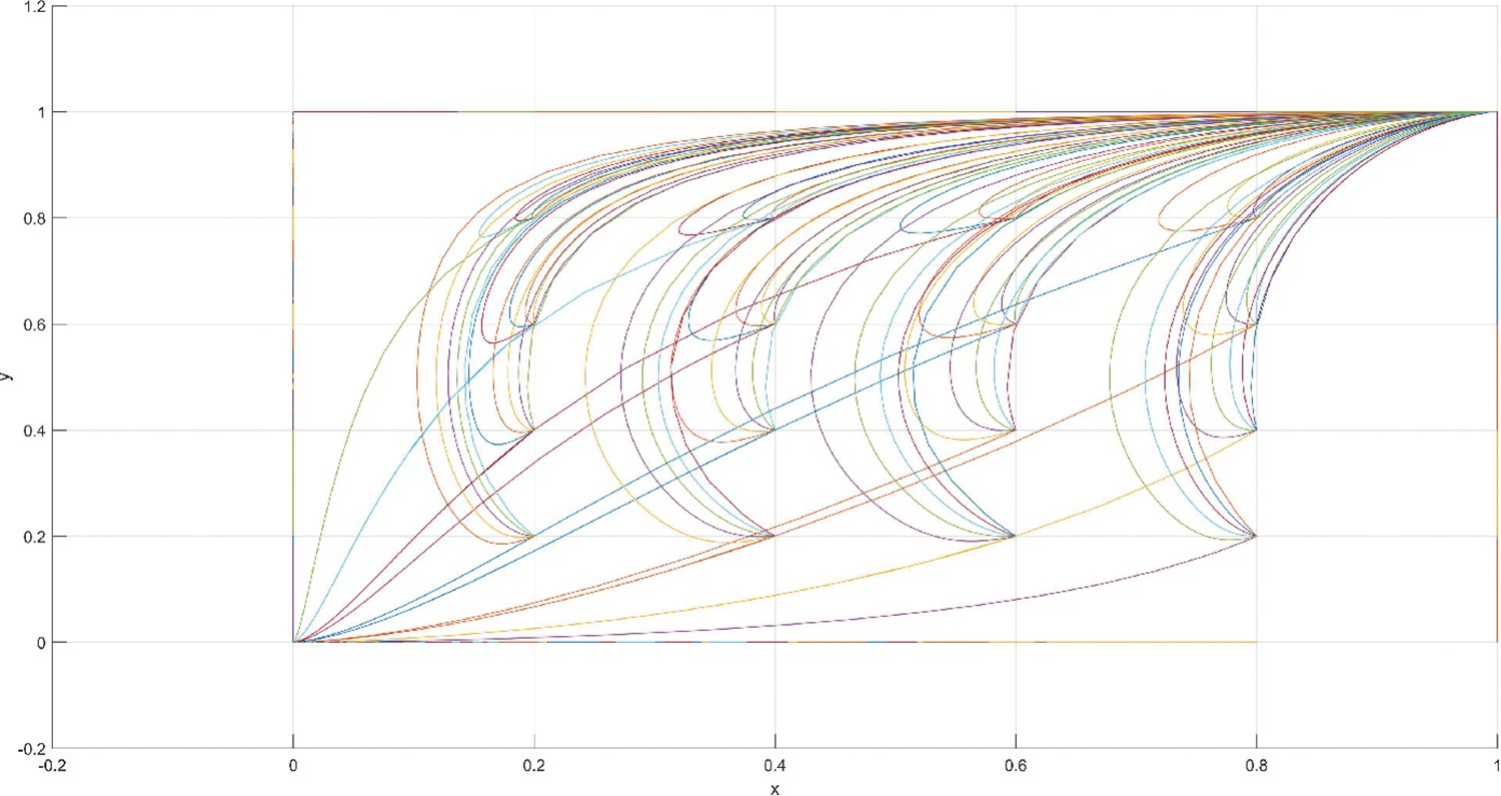
Scenario 4 x-y plane three-way evolutionary game diagram.

**Fig 17:**
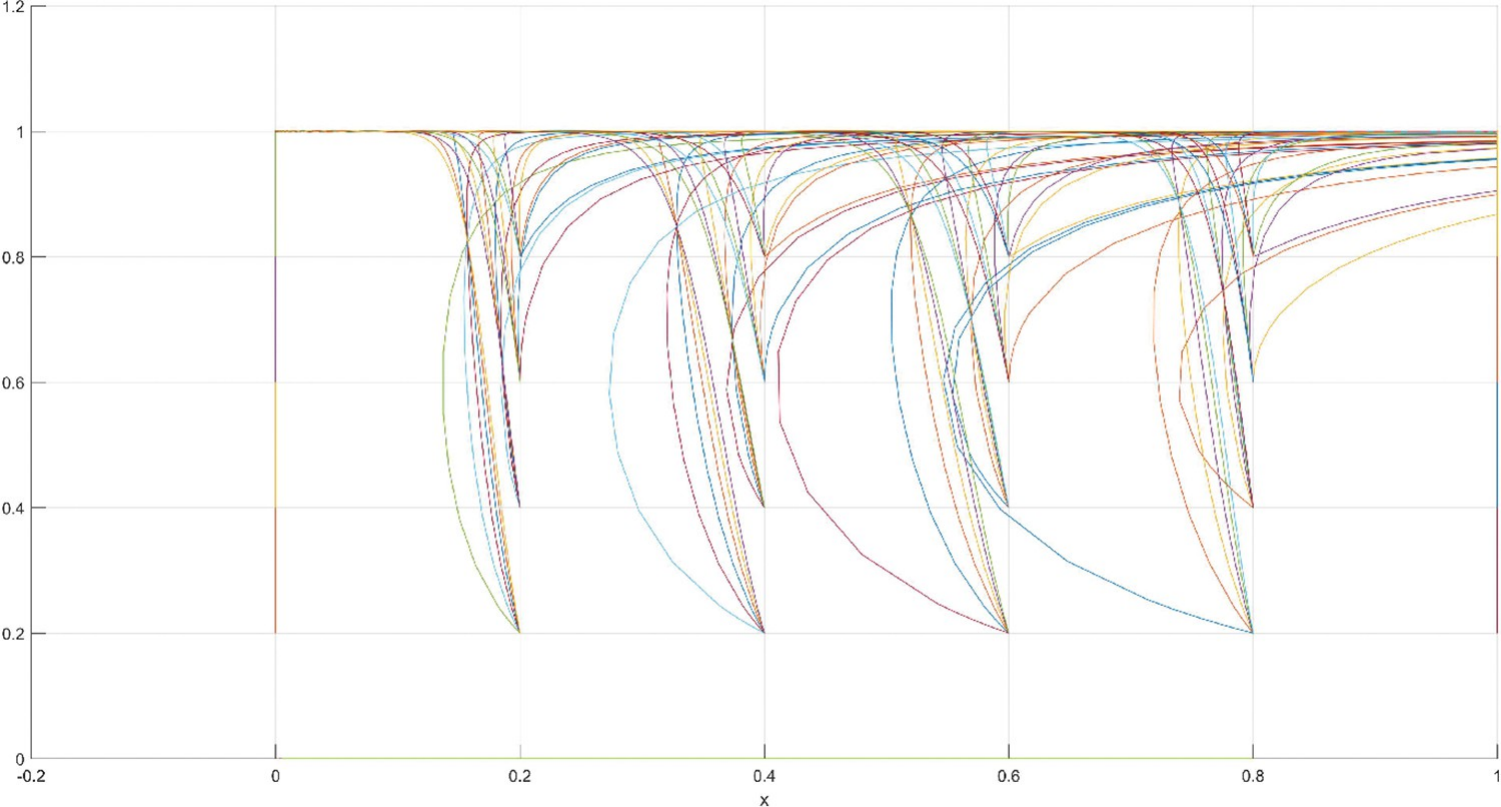
Scenario 4 x-z plane three-way evolutionary game diagram.

**Fig 18:**
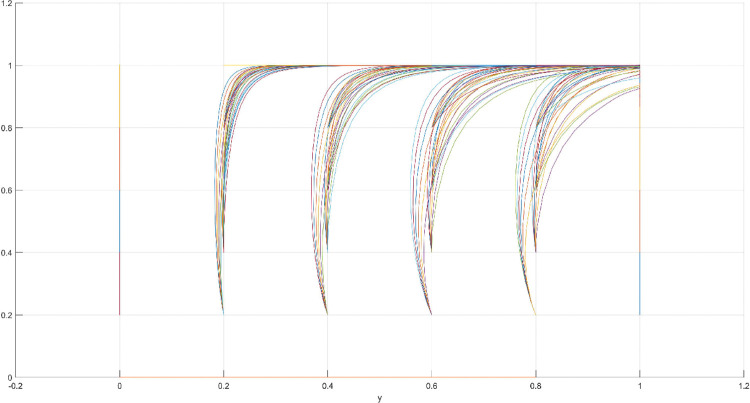
Scenario 4 y-z plane three-way evolutionary game diagram.

Scenario 4 is based on the conditions of leading firms’ cost of filing and maintaining the patent being less than the royalties or compensation received, and the government’s revenue from fines being greater than the sum of regulatory costs and patent subsidies. Similar to Scenario 3, the cost of substitution for following firms is assumed to be higher than the cost of imitation in addition to the royalties to be paid to the leading firms. The fines received by the government when it is regulating are greater than the patent subsidies and regulatory costs spent, and the government chooses to regulate. Based on the aforementioned analysis, the values of the parameters for Scenario 4 are assumed as follows: R = 100, S = 10, C1 = 50, C2 = 30, C3 = 20, R1 = 200, W = 50, δ = 0.5, α = 2, β = 4, μ = 2, and θ = 0.5. These values are substituted into the replication dynamic equation, and the evolutionary game diagram was drawn (Figs 15–18). x, y, and z evolve over time toward point E8 (1,1,1), which is consistent with the equilibrium point of the evolutionary game in the Jacobi matrix, corresponding to the measurement of the slight combination of {government regulation, patent application, imitation}.

According to the overall simulation results and because of finite rationality and exogenous environmental influence, the overall trend of simulation model results is consistent with the stable analysis of the dynamic replication equation. Moreover, the government, leading firms, and groups of following firms all tend to evolve stable analysis results after a certain period of game and mutual learning.

### Equilibrium point comparison analysis

A comparative analysis of the four scenarios was conducted on the basis of the above simulation analysis of the four equilibrium points (Table 5). As can be seen from the table, the difference between Scenario 1 and Scenario 2 is the difficulty of imitation and substitution of the innovation results of leading firms by following firms. The strategy choice of leading firms in Scenario 1 and Scenario 2 is technology secrecy; the strategy choice of the government is no regulation; and following firms develop a following strategy based on the comparison of imitation and substitution costs. This indicates that the most important factor affecting Scenario 1 and Scenario 2 is whether the innovation of leading firms is high-end and difficult to replicate. The conditional difference between Scenario 3 and Scenario 4 is the magnitude of government revenue from fines versus the cost of spending on regulation versus patent subsidies. The leading firms’ strategy in both Scenario 3 and Scenario 4 is to apply for a patent, while following firms choose an imitation strategy, and the government chooses a strategy based on comparing penalty revenues with the expenditure of regulation and patent subsidies. This indicates that the government makes penalty and subsidy policies that affect its own strategy choice.

**Table 5:** Comparative analysis of four scenarios.

Scenarios	Parameters	Conditions	Results
**1**	**Rθ < C2, β < α**	The patent infringement compensation obtained by leading firms is smaller than the cost of patent application and maintenance; the difficulty of replacing the innovation of leading firms by following firms is smaller than the difficulty of imitation.	Government chooses not to regulate, leading companies choose technology secrets, and following companies choose substitution
**2**	**Rθ<C2, α<β**	The patent infringement compensation obtained by leading companies is smaller than the cost of patent application and maintenance; it is more difficult for following firms to replace the innovations of leading firms than to imitate them.	The government chooses not to regulate, leading companies choose technology secrets, and following companies choose to imitate
**3**	**C2 < Rθ < C1β-C1α, W<C3+S**	The patent infringement damages received by leading firm are greater than the cost of patent application and maintenance; the cost of substitution by following firms is greater than the cost of imitation plus the payment of royalties to leading firms; and the government’s revenue from fines is less than the sum of regulatory costs and patent subsidies.	The government chooses not to regulate, leading companies choose to apply for patents, and following companies choose to imitate
**4**	**C2 < Rθ < C1β-C1α, C3+S<W**	The patent infringement damages received by leading firms are greater than the cost of patent application and maintenance; the cost of substitution by following firms is greater than the cost of imitation plus the payment of royalties to leading firms; and the government’s revenue from fines is greater than the sum of regulatory costs and patent subsidies.	The government chooses to regulate, leading firms choose to apply for patents, and following firms choose to imitate

## Discussion

### Conclusion

Enterprise innovation strategy selection is a hot topic of research. In practice, the regulation of government enterprises’ innovative behavior is being gradually improved; however, it is still far from extensive, adequate, and effective. The present study is based on the current development context of "mass entrepreneurship and innovation" in China and the strategic choice of innovation outcomes faced by leading and following firms. This study scrutinizes the heterogeneous effects of government regulatory tools and the difficulty of imitating innovation outcomes on firms’ innovation behavior under different conditions. Furthermore, the study helps promote innovation practices of Chinese firms and governmental regulatory reforms on firms’ innovation outcomes, in addition to providing useful references to guide the practical decision-making process for the sustainable development of innovation ecosystems, thereby providing a practical theoretical basis for the advancement of innovation goals. In addition, it has important practical significance and application value for fostering benign and sustainable competitive advantages among enterprises and improving innovation policies and market regulation system.

To achieve this goal, this study examines the technological strategy choice of subjects in the innovation ecosystem. First, it theoretically analyzes the dynamic game among the government and the leading and following enterprises by using evolutionary game theory to obtain the benefit matrix of the game and the system evolutionary stabilization strategy, proving insights into the technological strategy evolution process of innovation ecosystem subjects from an ecological perspective. Further, the assumptions of limited rationality and incomplete information are introduced, and the influencing factors and boundary conditions of the evolutionary game analysis are extended through data simulation to analyze the formation and evolution of the strategies of three subjects under different conditions. Based on the theoretical and simulation analyses, the following conclusions are drawn:

The cost of patent operation and maintenance, government subsidies, and the relative difficulty of technology substitution and imitation are the key factors affecting the evolutionary equilibrium of the system. Based on the different situations formed due to the aforementioned factors, four equilibria with different conditions emerge in the system: {no government regulation, technology secrecy, substitution}, {no government regulation, technology secrecy, imitation}, {no government regulation, patent application, imitation}, and {government regulation, patent application, imitation}.

The protection strategies of leading firms are influenced by the decisions of following firms. The same proportion of the leading enterprises’ two choices, namely patent application and technology, indicates that the difference between the two decisions brings slight benefit to the leading enterprises. The decisions affecting leading enterprises are mainly influenced by the decisions of following enterprises. The leading firms evaluate their decision according to the additional gain or loss brought by the decision of the following firms. As in the Jacobi matrix, when Rθ < C2, that is, the cost of patent application and maintenance for the leading firm is greater than the infringement compensation or patent license fee paid by the following firm, the leading firm chooses the technology secret decision.

A follower firm’s strategy choice is influenced by the leading firm’s protection strategy for the innovation as well as the government’s penalty policy. In addition, its strategy choice focuses on the difficulty of imitation or substitution of the innovation outcome. Among the constraints, when α < β, the leading firm’s innovation is of low technological content and does not require high human, financial and material resources; accordingly, the following firm can easily meet the conditions to imitate the production and save R&D costs. At this time, the following firm chooses the imitation strategy. On the contrary, when α > β, the leading enterprise innovation results with high technical content will require high resources such as talents, capital, and equipment, which are difficult to imitate, and thus, the following enterprise will choose the alternative strategy.

Government interventions ultimately have a direct impact on the government’s own benefits. The government regulation strategy has less influence on the choice of firms’ innovation decisions and is also minimally influenced by firms’ decisions. According to the four stable points of Jacobi matrix, the government chooses not to regulate E1 (0,0,0), E4 (0,0,1), and E7 (0,1,1) in three of the equilibrium decisions. This is because the way in which the government can gain throughout the dynamic game is the social benefits of innovation under the condition that the leading firm applies for a patent and the fine for following the firm’s infringement under this condition. Therefore, the government chooses the non-regulatory strategy considering the optimal gain. However, when the main purpose of the government is not to gain from it but to regulate the market equilibrium, it chooses to regulate.

### Managerial implication

First, this study adopts a dynamic and diverse set of strategies from the innovation ecosystem perspective. An evolutionary game model of the government and the leading and following firms was constructed, and the impact of tripartite behavior on the innovation ecosystem was investigated. Research in the area of corporate innovation and evolutionary games is theoretically supplemented. Second, this study integrates the mechanisms of patent system disclosure, protection, and subsidies in the context of China’s intellectual property system and firm management practices, and adds two variables of imitation and substitution difficulty of innovation outcomes. This is a new idea for exploring studies that affect firms’ behavioral strategies. Finally, two complementary research methods, namely dynamic evolutionary game and simulation, are used to enrich literature on the evolutionary mechanism of technology strategy formation of innovation ecosystem subjects and deepen the theory related to technology competition.

### Practical implications

Government interventions in innovation systems require context-specific policy measures. In the early stage of innovation ecological development, moderate incentives for corporate knowledge spillover and sharing are the basis for ecological scale growth and ecological niche diversification. In the middle stage of ecological development, optimizing the benign competition and cooperation relationship of the ecosystem through effective network governance means and avoiding opportunistic behaviors are the guarantee of ecological stability. In the late stage of ecological development, the key to ecological evolution is an open and inclusive innovation ecological environment that respects IPR and is proactive. The government should provide support to followers at the institutional and consciousness levels, stimulate followers to promote ecological growth through imitation, increase the overall knowledge output of society through substitution, stimulate innovation and entrepreneurship, and enhance enterprises’ independent innovation capacity [94].

Leading enterprises in the core position in the innovation ecosystem, as the guide, should adopt appropriate strategies at different stages of the innovation ecosystem development; maintain balance between exclusivity and sharing, scale and profit, short-term and long-term, and individual and network; maximize their own interests; and consolidate their core positioning and discourse power while promoting the development and transformation of the innovation ecosystem from a systemic and holistic view.

Leading enterprises need to consider the intensity of intellectual property protection, cost of dispute remedies, industrial environment, and technological characteristics; balance the relationship between knowledge advantages and knowledge spillovers; and build an innovation environment for the symbiotic and synergistic development.

Little attention has paid to the role of following firms in the innovation ecosystem and their strategies. Following enterprises play an irreplaceable role in the process of scaling, diversification, and evolution of innovation ecosystem. To grasp certain initiative in ecological development and the opportunity to become a core enterprise in ecological evolution, followers should focus on the balance between dependence on core enterprises and their own differences and independence, accelerate catching up through imitation, and form distinctive positioning through substitution.

Although this study has achieved the desired goal, its shortcomings should be acknowledged. First, the government’s function cannot be accurately assessed using revenue alone as a reference source for decision-making, and future research can consider other perspectives such as the value created. Second, this study uses the two-stage evolutionary game of three subjects, namely government, leading firms, and following firms, as the research model, combined with the conclusion of the study that following firms will make decisions based on the difficulty of imitating the innovation results of leading firms. Thus, a question arises that whether we can construct a three-stage game model for the three players. It is essential to explore whether the leading and following firms can change their relationship from being competitive to cooperative under the influence of the government’s decision-making behavior. In addition, industry-university-research has been a key issue in the field of innovation ecosystem, and the existing research revolves around the three promoting each other’s collaborative innovation. If the enterprise side is divided into the leading and following groups of enterprises, the collaborative innovation of the four subjects not only completes a perfectly closed loop among government, schools, and enterprises but also drives other enterprises to better innovation activities accordingly. Finally, the application of digital technology in fields, such as the key innovation areas of enterprises; application scenarios of innovative products; behavioral strategies of enterprises’ digital innovation results; the position and role of government and enterprises in the R&D of digital technology, should be highlighted in future research [95].

## Supporting information

S1 File (DOCX)
