# Supplementary material for: Government regulation strategy, leading firms’ innovation strategy, and following firms imitation strategy: An analysis based on evolutionary game theory
Source: PLoS One. 2023 Jun 8;18(6):e0286730. doi: 10.1371/journal.pone.0286730 (PMC10249874; doi:10.1371/journal.pone.0286730)
Supplement: S1 File — (DOCX) [file pone.0286730.s001.docx]

clc;clear;close all;

%%Assign values according to the actual situation

%%Scenario1 R=100;S=10;C1=50;C2=30;C3=20;R1=200;W=5;d=0.5;a=4;b=2;u=2;o=0.1;

%%Scenario2 R=100,S=10,C1=50,C2=30,C3=20,R1=200,W=5,d=0.5,a=2,b=4,u=2,o=0.1;

%%Scenario3 R=100,S=10,C1=50,C2=30,C3=20,R1=200,W=5,d=0.5,a=2,b=4,u=2,o=0.5;

%%Scenario4 R=100,S=10,C1=50,C2=30,C3=20,R1=200,W=50,d=0.5,a=2,b=4,u=2,o=0.5;

f = @(t,y)[y(1)*(y(1) - 1)*(C3 + S*y(2) - W*y(2)*y(3));

-y(2)*(y(2) - 1)*(S*y(1) - C2 + R*o*y(3));

y(3)*(y(3) - 1)*(C1*a*u - C1*b*u + C1*a*y(2) - C1*b*y(2) + R*o*y(2) + W*y(1)*y(2) - C1*a*u*y(2) + C1*b*u*y(2))];

for i=0:0.2:1

for j=0:0.2:1

for k=0:0.2:1

[t,y]=ode45(f,[0 1],[i;j;k;]);

% plot(t,y);

grid on

plot3(y(:,1),y(:,2),y(:,3));

hold on

end

end

end

hold off

% set(gca,'XTick',[0:0.2:1],'YTick',[0:0.2:1],'ZTick',[0:0.2:1])

xlabel('x');

ylabel('y');
